# Comparative Evaluation of Mutect2, Strelka2, and FreeBayes for Somatic SNV Detection in Synthetic and Clinical Whole-Exome Sequencing Data

**DOI:** 10.3390/biom15111532

**Published:** 2025-10-30

**Authors:** Igor López-Cade, Alicia Gómez-Sanz, Adrián Sanvicente, Cristina Díaz-Tejeiro, Aránzazu Manzano, Pedro Pérez-Segura, Balázs Győrffy, Alberto Ocaña, Miguel de la Hoya, Vanesa García-Barberán

**Affiliations:** 1 Experimental Therapeutics Unit, Oncology Department, Instituto de Investigación Sanitaria San Carlos (IdISSC), Hospital Clínico San Carlos (HCSC), 28040 Madrid, Spainalberto.ocana@salud.madrid.org (A.O.); 2 “Clinical and Translational Research in Oncology” Group, Molecular Oncology Laboratory, IdISSC, Hospital Clinico San Carlos, 28040 Madrid, Spain; 3 Department of Medical Oncology, IdISSC, Hospital Clínico San Carlos, 28040 Madrid, Spain; 4 Department of Medical Oncology, University Hospital 12 de Octubre, 28041 Madrid, Spain; 5 Research Institute [imas12], Complutense University of Madrid, 28040 Madrid, Spain; 6 Department of Bioinformatics, Semmelweis University, H-1094 Budapest, Hungary; 7 Cancer Biomarker Research Group, Institute of Molecular Life Sciences, HUN-REN Research Centre for Natural Sciences, H-1117 Budapest, Hungary; 8 Institute of Transdisciplinary Discoveries, Medical School, University of Pecs, H-7624 Pecs, Hungary

**Keywords:** variant caller, somatic, WES, variant allele frequency, read depth

## Abstract

Somatic variant calling is a critical step in cancer genome analysis, but the performance of available tools can vary depending on their underlying algorithms and filtering strategies. We compared three widely used variant callers—Mutect2, Strelka2, and FreeBayes—for their performance in somatic single-nucleotide variant (SNV) detection using both synthetic and real whole-exome sequencing (WES) data. Synthetic data were generated by introducing 4709 SNVs into a variant-free BAM file, while real data consisted of tumor and matched normal WES samples from five ovarian cancer (OC) patients. All callers were run using the nf-core/sarek pipeline with default settings and appropriate filtering. In the synthetic dataset, all tools showed high precision (~99.9%), with Mutect2 achieving the highest recall (63.1%), followed by Strelka2 (46.3%) and FreeBayes (45.2%). In real samples, FreeBayes detected the most variants, and only 5.1% of SNVs were shared across all three tools. We then integrated calls with SomaticSeq in consensus mode (Mutect2 + Strelka2) and kept variants with stronger allelic signals—showing higher VAFs and, typically, higher coverages relative to single-caller only. Caller-exclusive variants showed significant differences in allele frequency and sequencing depth. These results highlight substantial variability in SNV detection across tools. While all showed high specificity, differences in sensitivity and variant profiles underscore the need for context-specific caller selection or ensemble approaches in cancer genomics.

## 1. Introduction

WES is widely used in cancer research and clinical diagnostics to identify mutations in coding exons and nearby intronic sequences, which account for the majority of disease-associated variants [1]. This cost-effective approach enables large-scale molecular profiling and has been instrumental in initiatives such as The Cancer Genome Atlas (TCGA). In OC, WES has revealed key somatic alterations, including mutations in *TP53* and DNA repair genes such as *BRCA1* and *BRCA2*, which have direct implications for prognosis and treatment selection [2].

Detection of somatically acquired SNVs and small insertions or deletions (Indels) using WES data relies on variant callers (VCs) specifically designed for this purpose. These VCs typically accomplish their task by comparing sequencing data from tumor samples with matched normal samples (usually, blood-derived) from the same individual.

Among the many available tools, Mutect2, Strelka2, and FreeBayes are commonly used due to their strong performance and broad adoption [3]. Mutect2 [4], developed by the Broad Institute, employs haplotype reconstruction and Bayesian modeling, and tends to perform best for somatic mutations at variant allele frequencies (VAFs) higher than ~10%. Strelka2 [5], developed by Illumina, uses a position-wise probabilistic model with strict filters, favoring high-confidence calls, and has been shown to detect somatic mutations at lower VAF values, even down to ~5%. FreeBayes [6], although originally designed for germline variant detection [7], is often applied to tumor-only data due to its flexibility; it can report calls at VAF as low as ~0.01–0.05, albeit with a more permissive profile and increased false positive risk [3].

Prior comparative studies have demonstrated that these tools vary in sensitivity and specificity, depending on sample characteristics and variant allele frequency (VAF) [8]. Although synthetic benchmarking has become more common, many evaluations still rely predominantly on clinical data, where the absence of a ground truth complicates direct performance comparisons. Ensemble approaches have been proposed to enhance confidence, but consistent guidance on optimal caller combinations remains lacking [9]. Examples of such ensemble frameworks include SomaticSeq [10], which integrates multiple variant callers through a machine learning approach, and NeoMutate [11], which also applies supervised learning to features extracted from sequencing data. These ensemble strategies aim to increase precision and recall beyond what is achievable with individual tools, although they are not always easily accessible or standardized for routine use.

In this study, we systematically evaluate and compare the performance of Mutect2, Strelka2, and FreeBayes using both synthetic WES data (ground truth data), and real WES data from tumor-normal pairs of OC patients. We aim to evaluate key performance metrics such as recall and precision (in synthetic data) and to analyze concordance, variant characteristics, and potential downstream implications (in clinical samples). By integrating both types of datasets, we aim to provide practical guidance on VC selection and highlight the impact of caller-specific differences on downstream applications, such as mutational profiling and neoantigen prediction.

## 2. Materials and Methods

### 2.1. Patient Cohort

Five patients with OC were recruited from the Ovarian Cancer Unit at Hospital Clínico San Carlos. Detailed information about the patients is summarized in Supplementary Table S1. The study received approval from the Institutional Ethical Committee of Hospital Clínico San Carlos (approval number: 20/042-E_BS) and was conducted in accordance with Good Clinical Practice guidelines and the Declaration of Helsinki. Written informed consent was obtained from each participant. Diagnoses were confirmed through medical records and pathology reports.

### 2.2. DNA Extraction and Whole-Exome Sequencing

Tumor DNA was extracted from 4 to 8 sections of formalin-fixed paraffin-embedded (FFPE) tissues. A hematoxylin and eosin-stained slide from each sample was reviewed by a pathologist to determine tumor cell area and percentage. DNA extraction was performed using the GeneRead DNA FFPE Kit (Qiagen, Hilden, Germany) following the manufacturer’s protocol. Germline DNA (gDNA) was isolated from peripheral blood mononuclear cells (PBMCs) using the MagNA Pure Compact Nucleic Acid Isolation Kit (Roche Diagnostics, Grenzach-Whylen, Germany). DNA quantification was carried out using a Qubit v3.0 Fluorometer (Thermo Fisher Scientific, Waltham, MA, USA).

Libraries for WES were prepared with the SureSelect Human All Exon V6 kit (Agilent, Santa Clara, CA, USA), with at least 600 ng of input DNA. Library quality was verified using TapeStation D1000 (Agilent, Santa Clara, CA, USA). Paired-end sequencing (2 × 150 bp) was conducted on an Illumina NovaSeq platform. The average sequencing depth across SNV positions (based on the combined variant calls from the three tools) was approximately 294× in tumor samples and 120× in germline samples.

### 2.3. Generation of Synthetic Data with Known Somatic SNVs

Artificial datasets were generated using BAMSurgeon version 1.4.1 to create a controlled environment for evaluating VC performance [12]. A BED file was first generated containing 10,000 randomly selected SNVs from the COSMIC database [13]. Of these, 4709 SNVs were successfully introduced into the simulated data. The main reasons for unsuccessful insertions were insufficient or excessive local read depths outside the user-specified range of (50×–500×) and failure to meet BAMSurgeon’s internal requirements for variant incorporation (e.g., read pairing and alignment constraints).

Two artificial BAM files were generated: one representing a germline sample (with no introduced mutations) and another representing a tumor sample (containing the 4709 introduced SNVs).

BAMSurgeon was run with the following parameters:

--mindepth 50 --maxdepth 500 --minmutreads 5 --procs 60 --alignerthreads 32 --requirepaired --seed 1234

The inserted SNVs had a VAF ranging from 1% to 100%, with a mean VAF of 50%. The mean sequencing depth at variant positions was 99×.

### 2.4. Variant Calling and Bioinformatic Workflow

Raw FASTQ files were processed using the nf-core sarek pipeline version 3.5.0 with default parameters to provide a consistent baseline for benchmarking purposes [14,15]. Reads were aligned to the GRCh38 reference genome using BWA-MEM [16]. Somatic SNVs were identified independently by three VCs: Mutect2 (version 2.2), Strelka2 (version 2.9.10), and FreeBayes (version 1.3.6). Mutect2 and Strelka2 implement internal filtering criteria to discriminate high-confidence variants; therefore, only variants annotated with “PASS” in the FILTER field were retained from these callers. FreeBayes does not perform internal variant filtering; thus, variants identified by FreeBayes were subsequently filtered using the following criteria: QUAL ≥ 1, SAF > 0, and SAR > 0 (to ensure supporting reads on both DNA strands), and RPL > 1 and RPR > 1 (requiring at least two reads supporting the variant on both left and right flanks). No further variant annotation or external database filtering was performed; analyses were conducted directly on the filtered but unannotated variant call format (VCF) files.

### 2.5. Performance Metrics and Variant Comparison Strategy

Statistical analyses and data visualization were conducted using R version 4.4.2 within RStudio (version 2024.12.0 build 467). Principal R libraries utilized included ggplot2, dplyr, ggpubr, vcfr, and FSA for statistical evaluations and visualizations. Tests applied for comparisons included Kruskal–Wallis, Dunn’s post hoc test, and the Wilcoxon rank-sum test [17,18]. Plots were generated using customized ggplot2 themes.

Recall for synthetic data was evaluated based on the number of true positives (TP, variants present in both the truth set and the query callset) and false negatives (FN, variants present in the truth set but missed by the caller) (TP/[TP + FN]). Precision was calculated based on TP and false positives (FP, variants detected by the caller that are not present in the truth set) (TP/[TP + FP]). In this context, recall reflects the sensitivity of each caller, whereas precision relates to its specificity.

### 2.6. Ensemble Variant Calling Using SomaticSeq

We integrated single-caller results using SomaticSeq v3.7.0 in consensus mode (no machine-learning model). SomaticSeq was run on tumor–normal WES pairs using the same BAMs previously processed with nf-core/sarek, the GRCh38 reference, and the exome target BED, in paired mode. As inputs, we provided the PASS SNV VCFs from Mutect2 and Strelka2; FreeBayes calls were included only for overlap/descriptive comparisons, but were not used to assign confidence categories. In consensus mode, SomaticSeq inherits the callers’ filters and aggregates evidence across VCFs while extracting additional BAM-level features (e.g., read counts, mapping/strand metrics) to retain or discard candidates; no new fixed VAF/DP thresholds are introduced by SomaticSeq.

Substitution-type distributions were computed in R 4.4.2 (ggplot2), using the 12 REF > ALT classes and summarized as percentages per caller and for the SomaticSeq consensus set. Statistical comparisons used Kruskal–Wallis (global) and pairwise Wilcoxon tests with Benjamini–Hochberg adjustment; significance was set at *p* < 0.05.

## 3. Results

### 3.1. Variant Detection in Synthetic WES Dataset

**Figure 1:**
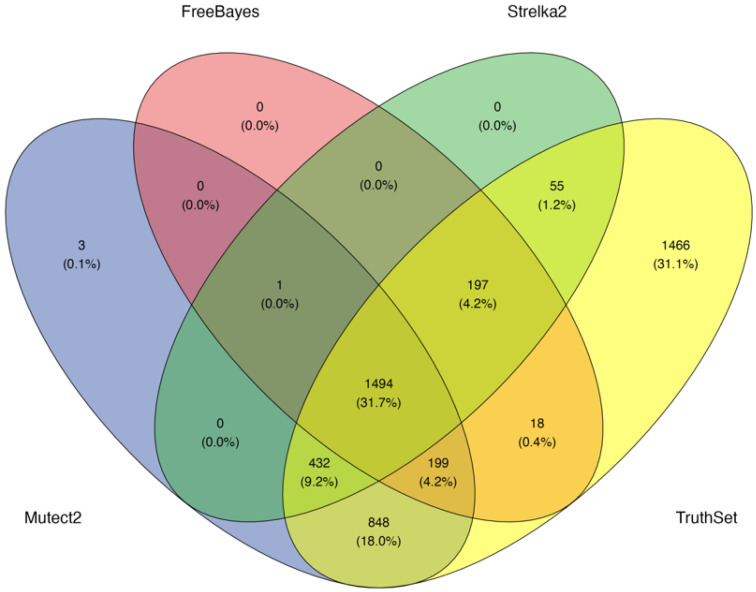
Overlap of detected SNVs among Mutect2, Strelka2, FreeBayes, and the ground truth set in the synthetic dataset.

A synthetic artificial dataset was generated introducing 4709 SNVs, which was used to analyze the performance of the three VCs, Mutect2, Strelka2, and FreeBayes. The overlap between the detected SNVs and the truth set is shown in Figure 1. A total of 31.7% (n = 1494) of the inserted variants were concordantly identified by all three callers. 18% (n = 848) of SNVs was detected only by Mutect2, 0.4% (n = 18) only by FreeBayes, and 1.2% (n = 55) only by Strelka2. Variants jointly detected by two tools included: 4.2% (n = 199) by Mutect2 and FreeBayes, 9.2% (n = 432) by Mutect2 and Strelka2, and 4.2% (n = 197) by FreeBayes and Strelka2. In contrast, 31.1% (n = 1466) of the inserted SNVs were not detected by any of the three callers, representing FN. Additionally, a small number of FPs were observed, including three unique to Mutect2 and one shared across all callers. All three callers demonstrated very high precision. Mutect2 achieved a precision of 0.9987, while FreeBayes and Strelka2 both reached 0.9995, indicating few FP calls. Mutect2 achieved the highest recall (63.1%), followed by Strelka2 (46.3%) and FreeBayes (45.2%). Strelka2 and FreeBayes showed higher FN rates compared to Mutect2 (Table 1).

To characterize the influence of VAF and sequencing depth (DP) on variant detection, we analyzed their distributions between TPs and FNs for each VC (Supplementary Figure S1). An enrichment of low-VAF variants was observed for FNs, particularly for Strelka2 and FreeBayes. For instance, approximately 45–50% of FN variants in both tools had VAF < 0.15, compared to only 13–17% of TPs. In contrast, Mutect2 showed a more balanced VAF distribution across TP and FN variants, although a modest excess of FNs was still observed in the lowest VAF bins.

Regarding sequencing depth, TP and FN variants for Mutect2 and Strelka2 showed similar distributions centered around log10(DP) ≈ 2 (i.e., ~100×). However, in the FreeBayes results, FN variants exhibited a visible shift toward lower depths, with many FNs clustering around log10(DP) < 1.8 (~63×), whereas TPs peaked closer to 100×. These patterns suggest that FreeBayes is less sensitive to low coverage, while Strelka2 and Mutect2 are more affected by low allele frequency.

To further evaluate caller performance on synthetic data, we analyzed the distribution of mutation types (REF→ALT substitutions) detected by each VC and compared them with the baseline set of mutations introduced by BamSurgeon (Supplementary Figure S2). As expected, the majority of substitutions corresponded to C > T and G > A transitions, which were also the most frequently detected by all three callers. Mutect2 reported slightly higher frequencies of these common transitions compared to Strelka2, while FreeBayes showed intermediate results. Despite these caller-specific tendencies, all three tools reproduced the mutational spectrum introduced in the synthetic dataset with only minor differences, suggesting that the variations observed reflect intrinsic characteristics of each caller.

### 3.2. Detection of Somatically Acquired Variants in Ovarian Cancer WES Samples

**Figure 2:**
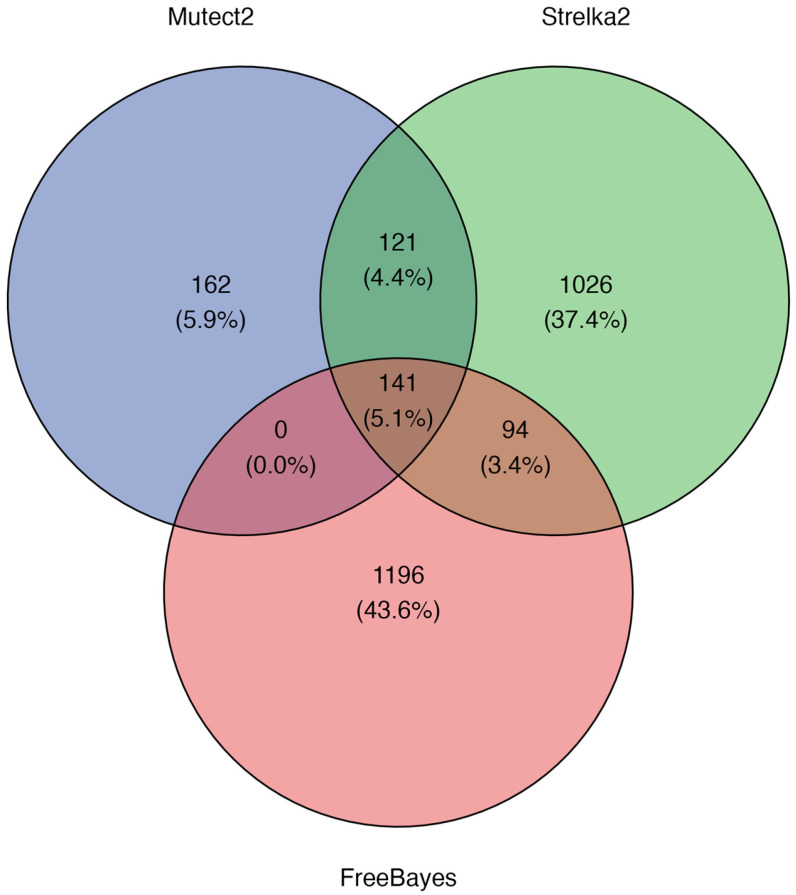
Overlap of somatic SNVs detected by different VCs. Overlap of somatic SNVs detected by Mutect2 (blue), Strelka2 (green), and FreeBayes (red) in real WES somatic SNVs from five OC patients.

To assess VC performance in a clinical context, we analyzed somatically acquired SNVs detected by the three VCs in WES data from five OC tumor samples. The analysis was restricted to SNVs, as only this type of variant was introduced in the synthetic dataset across all samples. Moreover, indels were excluded due to the substantial complexity they introduce in benchmarking analyses. Accurately comparing indels requires stratifying results based on multiple parameters, such as indel length, insertion versus deletion, or sequence context, which can lead to inconsistent metrics and hinder robust cross-tool evaluation. Focusing on SNVs allowed for a more controlled and interpretable comparison across callers. Percentages were calculated based on the total number of unique variants identified across all samples. Mutect2, Strelka2, and FreeBayes detected a total of 424, 1382, and 1431 SNVs, respectively. The overlap among the SNVs detected by each VC is shown in Figure 2. FreeBayes identified the highest number of unique variants (43.6%, n = 1196), followed by Strelka2 (37.4%, n = 1026), and Mutect2, which detected significantly fewer variants (5.9%, n = 162). Shared variants were less frequent: 4.4% (n = 121) were detected by both Mutect2 and Strelka2, 3.4% (n = 94) by Strelka2 and FreeBayes, and only 5.1% (n = 141) were consistently identified by all three tools.

### 3.3. Differences in DP and VAF Between Exclusive and Shared Variant Calls in Ovarian Cancer WES Samples

To further characterize these calls, we examined the VAF and DP distributions for all detected SNVs with each VC. The VAF distribution was skewed toward low-frequency variants, while sequencing DP was centered around ~180×, calculated as the average of the median DP values reported by the three callers (170× for Mutect2, 137× for Strelka2, and 231× for FreeBayes). This value is consistent with expectations for high-coverage WES data (Supplementary Figure S3).

**Figure 3:**
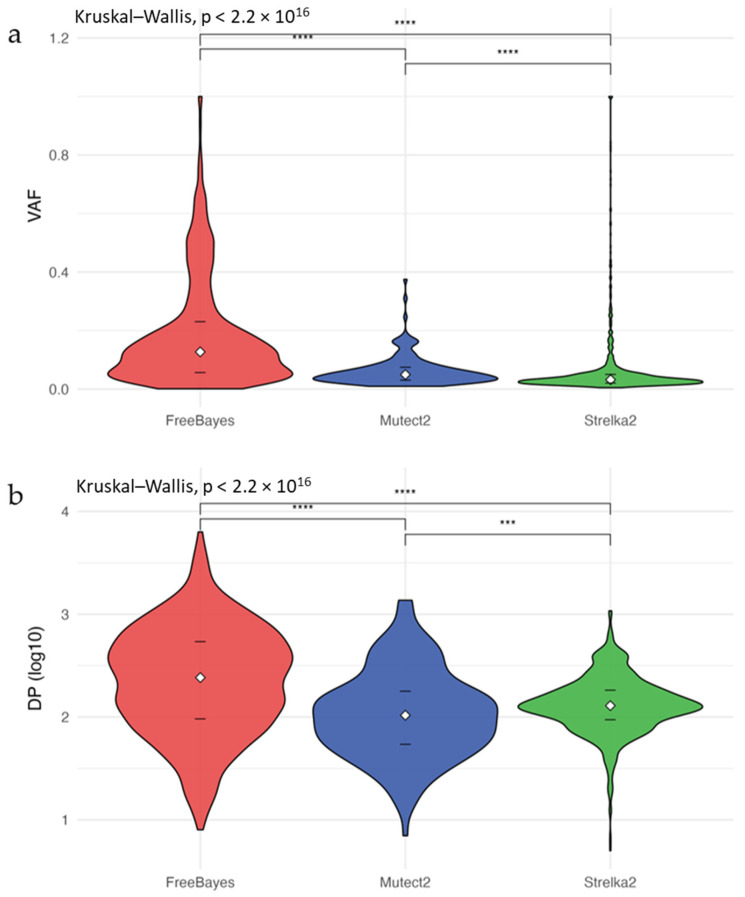
Distribution of (**a**) VAF and (**b**) DP for exclusive SNVs detected by Mutect2, Strelka2, or FreeBayes. White diamonds represent medians; black lines indicate Q1 and Q3. Horizontal bars show Wilcoxon tests; Kruskal–Wallis *p*-values appear in the upper left. Significance: *** *p* < 0.001, **** *p* < 0.0001.

Moreover, VAF (Figure 3a) and DP (Figure 3b) of SNVs uniquely identified by each caller in the patient WES data were analyzed. Among the exclusive variants, FreeBayes showed the highest median VAF (0.13) and depth (242), followed by Mutect2 (median VAF = 0.05, depth = 104) and Strelka2 (median VAF = 0.032, depth = 129). These differences were statistically significant for both VAF and DP (Kruskal–Wallis *p* < 0.05; Dunn’s test *p* < 0.001 for among exclusives). In contrast, shared variants detected by all three callers did not exhibit significant differences in VAF or DP (Wilcoxon and Kruskal–Wallis *p* > 0.05). These findings are summarized in Supplementary Tables S2 and S3 and Supplementary Figure S4.

### 3.4. Ensemble Concordance and Caller-Resolved VAF/DP Profiles

We re-called SNVs with SomaticSeq in consensus mode over Mutect2 and Strelka2 and stratified PASS variants into six groups: (i) FreeBayes exclusive; (ii) Mutect2 only; (iii) Mutect2∩Strelka2 only; (iv) Mutect2∩Strelka2∩SomaticSeq; (v) Strelka2 exclusive; and (vi) Strelka2∩SomaticSeq. The overlap among all PASS sets from the three individual callers and SomaticSeq is shown in Supplementary Figure S5. VAF and DP per group were compared (Supplementary Figure S6 and Supplementary Table S4a). SNVs detected by Mutect2∩Strelka2∩SomaticSeq exhibited significantly higher VAFs than SNVs called by Mutect2∩Strelka2, Mutect2 only, or Strelka2 only; median VAFs were 0.167, 0.031, 0.049, and 0.033, respectively. (Supplementary Table S4b). SNVs identified by multi-caller consensus sets had significantly higher coverage than those detected by a single caller: median DP 228.5 for Mutect2∩Strelka2∩SomaticSeq, and 169 for Mutect2∩Strelka2 versus 129 for Strelka2 only and 104 for Mutect2 only (Supplementary Table S4c). All group comparisons for both VAF and DP metrics are shown in Supplementary Table S4.

Finally, the substitution-type distribution for the SomaticSeq consensus shows the expected predominance of C > T/G > A transitions (Supplementary Figure S2).

## 4. Discussion

This study aimed to evaluate the performance of SNV detection across three VCs, Mutect2, Strelka2, and FreeBayes, by applying them to both synthetic and real WES data. A synthetic dataset was generated to provide a ground truth for benchmarking, enabling a controlled evaluation of precision and recall. Of note, the synthetic data simulated somatically acquired variants, but did not include any germline background of genetic variability, allowing for a direct assessment of somatic variant detection but limiting evaluation of false positives arising from germline contamination. The real dataset consisted of WES from five ovarian tumors, allowing a comparative analysis of the number and characteristics of variants identified by each tool in a clinically relevant context. While performance metrics such as sensitivity and precision were only quantifiable on the synthetic data, the analysis of caller-exclusive and shared variants in patient samples offered complementary insights into the behavior of each tool under real-world conditions.

Among the three VCs in the synthetic benchmark, Mutect2 exhibited the most favorable balance between sensitivity and precision, achieving the highest recall while maintaining strong precision. All three VCs achieved very high precision (~99.9%), indicating that FPs were rare across all tools. This suggests that under ideal conditions with clean data, Mutect2, Strelka2, and FreeBayes implement effective filters to minimize spurious calls. However, recall values were notably lower, revealing substantial differences in sensitivity. Mutect2 detected approximately 63% of the known synthetic variants, while Strelka2 and FreeBayes detected around 46% and 45%, respectively. In other words, over one-third of the true variants were missed by Mutect2, and more than half were missed by Strelka2 and FreeBayes. In total, over 30% of simulated variants were missed by all callers, highlighting a common limitation in somatic SNV detection.

The lower recall observed for Strelka2 and FreeBayes may reflect stricter filtering thresholds or a more conservative approach that prioritizes high-confidence variants, potentially excluding SNVs with low VAF. In contrast, Mutect2’s higher recall may be partially explained by its ability to detect low-frequency or low-DP variants more effectively. Although a custom panel of normals (PoN) was not explicitly specified, the Sarek pipeline includes a default PoN for the GATK.GRCh38 reference genome, which was automatically applied. This PoN consists of aggregated sequencing data from normal samples and is used by Mutect2 to filter out recurrent technical artifacts and sequencing noise. Its application likely contributed to the reduction in spurious calls.

It is worth noting that, given the design of the synthetic dataset, essentially no false positives are expected, which reinforces the idea that the observed discrepancies between tools reflect differences in sensitivity rather than specificity. Undetected variants for all VCs were more frequent in regions with low VAF, and this was also observed for FreeBayes in regions with low sequencing depth. These findings support the notion that sensitivity decreases significantly for low-frequency variants and that read support is a critical limiting factor for variant detection.

In the patient WES data, the three callers produced notably different numbers of somatic SNV calls. FreeBayes identified the highest number of variants across the five OC exomes, followed by Strelka2, while Mutect2 reported substantially fewer variants. These differences align with the expected behavior based on the design of each caller: FreeBayes, often regarded as more permissive, indeed showed a broader calling profile in this dataset. Strelka2, known for prioritizing high-confidence variants, produced fewer calls than FreeBayes but more than Mutect2. Mutect2, which applies stringent statistical filters and utilizes both a matched normal sample and a PoN, reported the smallest number of somatic SNVs.

The limited overlap between the callers, particularly the low proportion of variants shared by all three, reflects the known variability among somatic VCs [11,19]. By evaluating both synthetic data with a known ground truth and real tumor samples, our study provides complementary insights into how this discordance manifests across controlled and clinical settings. These differences align with prior benchmarking efforts, which show that while Mutect2 and Strelka2 often produce overlapping somatic SNV call sets, the tools differ in reproducibility and variant calling results, depending on the alignment strategy and pipeline configuration [20,21]. Our data extend this observation by including FreeBayes, showing that it produced the highest number of variants, including many exclusive calls not detected by the other tools. Despite not being traditionally used for somatic SNV calling, FreeBayes demonstrated reliable performance in our synthetic dataset, with high precision. However, its lower recall indicates that it missed a substantial proportion of true variants under controlled conditions, likely due to reduced sensitivity to low-frequency alleles and reduced coverage. In the synthetic dataset, Mutect2 and Strelka2 produced high-precision calls, but missed more low-frequency variants. The divergences between VCs suggest that each tool captures a distinct subset of the exome’s mutational landscape, reinforcing the importance of multi-caller approaches in both clinical and exploratory genomic analyses. Previous studies [22] recommend using multiple callers in parallel to mitigate caller-specific limitations and increase detection sensitivity, especially for clinically relevant mutations with low allele fractions.

### 4.1. Ensemble Integration with SomaticSeq

Comparison by caller reveals that SomaticSeq enriches for variants with stronger supporting evidence. The Mutect2∩Strelka2∩SomaticSeq set exhibited higher median VAF and DP than the Mutect2-only and Strelka2-only subsets, consistent with a consensus model that prioritizes robust signals. Further, within the Mutect2∩Strelka2 group, VAFs were lower than in the SomaticSeq consensus, while DP did not differ significantly. These results indicate that SomaticSeq rejects a subset of Mutect2 + Strelka2 overlaps when the allelic signal is weak despite comparable coverage, favoring precision over sensitivity in that range.

In addition to the 236 SNVs shared by Mutect2∩Strelka2∩SomaticSeq and Mutect2∩Strelka2, SomaticSeq also recovered SNVs called by Strelka2 but missed by Mutect2; these showed higher VAFs than the Strelka2-only set with no significant difference in DP, indicating the consensus favors allelic-signal quality and coherence (plus auxiliary quality features) over raw depth. For the Strelka2∩SomaticSeq set, VAF and DP did not differ significantly from Mutect2-only variants, implying SomaticSeq’s retention here reflects other model features such as base and read quality, strand-orientation patterns, soft-clipping, and local mappability rather than VAF or DP alone.

FreeBayes-exclusive variants showed high VAF and high DP, were not reported by Mutect2 or Strelka2, and were therefore absent from the SomaticSeq consensus.

This discrepancy likely reflects differences in caller modeling and filters—mapping-quality cutoffs, strand-bias rules, treatment of repetitive or low-complexity regions, local-assembly algorithms, and somatic-specific filters such as PoN usage, contamination models, and VAF priors. In other words, many high-coverage calls fail in certain somatic tools because of contextual or quality constraints rather than insufficient read depth.

Collectively, the results show that SomaticSeq mitigates caller discrepancies by preferentially retaining variants with higher VAFs and superior quality metrics while maintaining the expected mutational spectrum. This behavior explains why some caller-exclusive subsets—notably FreeBayes-only calls—are not included: their exclusion reflects divergent quality and contextual filters across callers rather than insufficient read depth.

C > T/G > A transitions were the most frequent; while a fraction may reflect residual FFPE-related deamination despite UNG treatment (which mitigates cytosine→uracil but not 5mC→T), these changes are not necessarily artifactual and should not be over-attributed to damage.

### 4.2. Clinical and Bioinformatic Implications

Our findings have several implications for downstream analyses in cancer genomics and clinical decision-making. One notable example is neoantigen prediction pipelines for cancer immunotherapy [23], which rely on somatic variant calls to identify mutant peptides [24]. In this analysis type, the initial call set strongly influences downstream prioritization. A conservative caller like Strelka may fail to detect many true somatic variants, potentially omitting immunogenic mutations from the prediction phase. Mutect2 demonstrated the best balance between recall and precision in our artificial analysis, making it suitable for contexts where both sensitivity and specificity are important, but it produced a lower number of variants in our real dataset. In contrast, although FreeBayes showed high precision, its lower recall suggests it may miss true variants unless parameter tuning or post-processing is applied. If not filtered properly, FPs, especially in callers with less stringent default settings, could lead to wasted effort or misleading targets in personalized vaccine or T-cell therapy design [25].

This highlights the need to tailor variant calling strategies to specific goals—whether maximizing sensitivity, as in neoantigen discovery, or prioritizing specificity for clinical decision-making. For instance, when identifying mutations related to drug resistance or selecting actionable alterations for targeted therapies, a conservative calling strategy may be preferred to avoid FPs. In contrast, exploratory settings like immunogenomics or early biomarker discovery may benefit from more sensitive or multi-caller approaches. Another key point for defining the calling strategies is tumor heterogenous. Highly heterogeneous tumors may harbor more low-VAF variants that conservative callers fail to detect, amplifying disparities across tools. Therefore, the context of the tumor and sequencing characteristics, and the clinical applications should be taken into account.

Moreover, the statistically significant differences in DP and VAF for each caller’s exclusive variants indicate that each tool has a bias toward detecting variants within specific ranges of sequencing depth and allele frequency. FreeBayes-exclusive variants tended to exhibit higher VAF and DP, while those uniquely identified by Strelka2 showed lower values for both metrics. These patterns suggest that taking the union of all variant calls maximizes sensitivity by capturing a broad spectrum of allele frequencies and sequencing contexts. However, restricting to variants detected by all three callers can exclude biologically relevant mutations, particularly those with low VAF. A balanced strategy would be to retain variants detected by at least two callers, which helps reduce FNs while preserving confident variants supported by both conservative and sensitive detection approaches. An awareness of these characteristics is important when designing downstream analyses. For example, based on our patient WES data, if one is performing mutational burden calculations or identifying driver mutations for pathway analysis [26,27], using only Mutect2 might underestimate the tumor mutational burden. Similarly, in a clinical bioinformatics pipeline, one might choose to run multiple callers and flag variants only detected by a single tool for further review. However, given the potentially large number of such unique calls, especially from more permissive callers, manual review (e.g., inspection in a genome browser) becomes impractical at scale. This highlights the need for automated filtering strategies to prioritize which unique variants may warrant follow-up validation [23]. Ensemble approaches (i.e., combining data from multiple VCs) or variant prioritization frameworks can help address these discrepancies by incorporating annotation data or confidence scoring.

### 4.3. Limitations

BAMSurgeon inserts mutations into sequencing data in silico, which guarantees a known truth set but cannot fully simulate challenging contexts such as complex genetic variants or sequencing artifacts that occur in actual tumor samples [28]. Therefore, recall and precision measured on the synthetic data might not translate exactly to real-world performance. For instance, real tumors often have subclonal populations and sequencing noise (due to FFPE DNA damage or other factors) that could affect caller performance differently than in a clean synthetic spike-in scenario.

In addition, previous studies have shown that even different versions of the same variant caller, such as Mutect2, can produce divergent results due to ongoing updates and algorithm refinements [29]. This highlights that variant calling performance is not static, and benchmarking results can shift over time as tools evolve.

A further limitation is that the ground truth in the patient data is unknown. We did not have orthogonal validation (such as deep amplicon sequencing) to confirm which caller’s unique variants were TPs or FPs. This limitation is common to many previous studies, which often rely on indirect evidence or consensus across tools to infer variant reliability. Furthermore, it is possible that some variants uniquely detected by FreeBayes represent residual germline variants that were not properly filtered out, whereas Mutect2 may have excluded them due to its built-in germline filtering using the matched normal. Thus, we cannot definitively determine whether additional variants called by FreeBayes are real somatic mutations missed by Mutect2, artifacts, or germline variants not filtered-out properly. This uncertainty underscores the need for cautious interpretation of individual caller outputs and supports the use of validation strategies or consensus-based approaches when feasible.

We also note the small number of tumor-normal WES samples analyzed (five ovarian cancer patients), which restricts the generalizability of our findings. In addition, orthogonal validation of variants in the real data was not feasible due to the low allele frequencies of many events and the cost of targeted resequencing assays. These aspects should be addressed in future work with larger patient cohorts and complementary validation strategies.

Finally, in this study, we applied the nf-core/sarek pipeline using the default parameters as described by the developers and in recent publications. While this choice ensured consistency and comparability, it may have affected the sensitivity of variant detection. Parameter optimization or customized filtering strategies could potentially reduce the number of false negatives and further improve the overall performance of the VCs.

### 4.4. Future Directions

A logical extension of this work would be to validate the findings across a broader cohort encompassing diverse tumor types and mutational landscapes. Rather than focusing solely on downstream applications, future work should address upstream sources of variability, particularly at the variant calling stage, as these initial discrepancies can substantially impact subsequent analyses such as neoantigen prediction or clinical variant interpretation.

Given the complementary strengths of the three tools examined, an important area to explore is ensemble or consensus variant calling strategies. Our results reinforce prior recommendations to combine VCs to mitigate tool-specific limitations. For example, one could imagine a voting scheme where a variant called by at least two out of three tools is accepted, or an advanced ensemble algorithm that learns from a truth dataset which caller’s output to trust in various scenarios. Care must be taken, as union-of-callers approaches increase sensitivity at the expense of more FPs, while intersection-of-callers approaches improve precision but can miss real variants. Recent ensemble frameworks have shown promise in improving accuracy by integrating multiple VCs [30]. In summary, future studies should aim not only to compare VCs across more samples and tools but also to integrate them in smart ways, ultimately striving for a robust, consensus-driven variant calling pipeline that can be confidently used in research and clinical settings.

## 5. Conclusions

This study presents a comparative evaluation of three widely used somatic SNV callers: Mutect2, Strelka2, and FreeBayes, using both synthetic and real WES data. While all tools demonstrated high precision, we observed meaningful differences in recall, call counts, and allele-frequency distributions, indicating that each caller captures a distinct subset of variants with potential impact on downstream analyses. Given the limited overlap among callers, strict intersection approaches are likely to sacrifice sensitivity.

Rather than endorsing a single best caller, our results support context-aware strategies, including ensemble integration. In our data, SomaticSeq (consensus over Mutect2 + Strelka2) increased concordance beyond the three-way overlap and preferentially retained variants with stronger allelic signal (higher VAF) without requiring higher depth, while preserving the expected C > T/G > A substitution profile. FreeBayes only subset showed high VAF and depth but was not retained by the consensus, likely due to divergent quality/context filters across engines (e.g., mapping quality, strand bias, local assembly). Ensemble methods offer a trade-off between precision and sensitivity and clarify the quality features driving call retention or rejection.

## Figures and Tables

**Table 1:** Summary of variant calling performance for each caller. Total Truth indicates the number of synthetic variants introduced. Total Query refers to the total number of variants called. TP: variants found in both truth and query sets; FP: variants called only in the query; FN: variants present only in the truth set.

Caller	Total Truth	Total Query	TP	FP	FN	Recall	Precision
Mutect2	4709	2977	2973	4	1736	0.6313	0.9987
FreeBayes	4709	1909	1908	1	2314	0.4519	0.9995
Strelka2	4709	2179	2178	1	2531	0.4625	0.9995

## Data Availability

The datasets generated and analyzed during this study—including the synthetic data, raw sequencing files (FASTQ), and all analysis scripts/code—are available from the corresponding author upon reasonable request. The datasets presented in this article are not readily available because they are part of an ongoing study.

## Supplementary Materials

The following supporting information can be downloaded at: https://www.mdpi.com/article/10.3390/biom15111532/s1, Figure S1: Distribution of VAF and log_10_-transformed sequencing DP of detected and undetected variants for each VCs in synthetic WES dataset. Detected true positives (shown in green) and undetected (false negatives, shown in red) are displayed separately; Figure S2: Distribution of single-nucleotide substitution types in OC WES data. Substitution spectrum detected independently by Mutect2, Strelka2, FreeBayes, and SomaticSeq consensus variants; Figure S3: Distribution of VAF and DP for SNVs detected by FreeBayes, Mutect2, and Strelka2 in OC WES data. The white diamond indicates the median, while the horizontal black lines represent the first (Q1) and third (Q3) quartiles. Horizontal bars denote pairwise Wilcoxon rank-sum tests. Kruskal-Wallis test *p*-values are shown in the upper left corner of each panel. Significance codes: *** *p* < 0.001, **** *p* < 0.0001; Figure S4: Distribution of VAF and DP for shared SNVs detected by FreeBayes, Mutect2, and Strelka2 in OC WES data. The white diamond indicates the median, while the horizontal black lines represent the first (Q1) and third (Q3) quartiles. Horizontal bars denote pairwise Wilcoxon rank-sum tests. Kruskal-Wallis test *p*-values are shown in the upper left corner of each panel. Significance codes: ns = not significant; Figure S5: Four-set Venn diagram by Mutect2, Strelka2, FreeBayes, and SomaticSeq (consensus mode). Each region shows the count and percentage of variants relative to the union across callers. Colors correspond to the four callsets (Mutect2: blue; Strelka2: green; FreeBayes: red; SomaticSeq: orange). Only SNVs are included; Figure S6: Violin plots summarizing (a) VAF and (b) DP (log10 scale) for SNVs partitioned into six groups: Mutect2-only, Strelka2-only, FreeBayes-only, Mutect2∩Strelka2only, Mutect2∩Strelka2∩SomaticSeq, and Strelka2∩SomaticSeq-only. Boxes indicate the median and interquartile range within each violin; Table S1: Clinical and molecular characteristics of the OC samples analyzed. The table includes age, histology, grade, FIGO stage, germline BRCA status (gBRCA), and tumor purity. “NA” indicates that the data was not available; Table S2: Pairwise comparisons of VAF and DP among exclusive SNVs detected by each VC using Dunn’s post-hoc test following a significant Kruskal-Wallis result; Table S3: Statistical comparison of VAF and DP between variant callers for shared variants. Wilcoxon rank-sum tests were used for pairwise comparisons, and Kruskal-Wallis tests were applied when more than two groups were analyzed; Table S4: (a) Descriptive statistics for SNVs by ensemble group: sample size (n), median and IQR of VAF and DP, range (min–max), and median log10(DP). (b) Pairwise Wilcoxon tests for VAF (two-sided; BH-adjusted *p*-values) with the overall Kruskal–Wallis *p*-value. (c) Pairwise Wilcoxon tests for log10(DP) (two-sided; BH-adjusted *p*-values) with the overall Kruskal–Wallis *p*-value. Statistical significance was defined as *p* < 0.05.

## Abbreviations

The following abbreviations are used in this manuscript:

BAM	Binary Alignment/Map
BWA-MEM	Burrows-Wheeler Aligner Maximal Exact Matches
CNV	Copy Number Variation
COSMIC	Catalogue Of Somatic Mutations In Cancer
DP	Read Depth
FFPE	Formalin-Fixed Paraffin-Embedded
FN	False Negative
FP	False Positive
GATK	Genome Analysis Toolkit
gDNA	Germline DNA
GRCh38	Genome Reference Consortium Human build 38
OC	Ovarian Cancer
PBMCs	Peripheral Blood Mononuclear Cells
PoN	Panel of Normals
RPL	Reads supporting the variant from the left side
RPR	Reads supporting the variant from the right side
SNV	Single Nucleotide Variant
TCGA	The Cancer Genome Atlas
TP	True Positive
VAF	Variant Allele Frequency
VC	Variant Caller
VCF	Variant Call Format
WES	Whole-Exome Sequencing
