# Supplementary material for: Comparative Evaluation of Mutect2, Strelka2, and FreeBayes for Somatic SNV Detection in Synthetic and Clinical Whole-Exome Sequencing Data
Source: Biomolecules. 2025 Oct 30;15(11):1532. doi: 10.3390/biom15111532 (PMC12650410; doi:10.3390/biom15111532)
Supplement: Supplementary file 1 [file biomolecules-15-01532-s001.zip › Supplementary Tables_round2.pdf]

Supplementary Table S1. Clinical and molecular characteristics of the OC samples analyzed. The table includes age, histology, grade, FIGO stage, germline BRCA status (gBRCA), and tumor purity. "NA" indicates that the data was not available.

Sample	Age	Histology	Grade	FIGO Stage	gBRCA	Purity
HCSC01	62	serous	High-grade	IIIC	NA	30%
HCSC02	63	serous	High-grade	IV	mut BRCA1	40%
HCSC03	80	serous	High-grade	IIIC	NA	60%
HCSC04	68	mucinous	High-grade	IIIC	NA	45%
HCSC05	86	germ cell	adult granullosa	IA	NA	30%

Supplementary Table S2. Pairwise comparisons of VAF and DP among exclusive SNVs detected by each VC using Dunn's post-hoc test following a significant Kruskal-Wallis result.

**Comparison**	**Metric**	**Z-score**	***p-value****	***Adjusted p-value****
FreeBayes Only vs. Mutect2 Only	VAF	9.40	5.43×10^{-21}	8.14×10^{-21}
FreeBayes Only vs. Strelka2 Only	VAF	26.49	1.19×10^{-154}	3.58×10^{-154}
Mutect2 Only vs. Strelka2 Only	VAF	4.03	5.69×10^{-5}	5.69×10^{-5}
FreeBayes Only vs. Mutect2 Only	DP	8.75	2.13×10^{-18}	3.20×10^{-18}
FreeBayes Only vs. Strelka2 Only	DP	12.38	3.18×10^{-35}	9.55×10^{-35}
Mutect2 Only vs. Strelka2 Only	DP	–2.43	1.50×10^{-2}	1.50×10^{-2}

*p-values < 0.05 were considered statistically significant.

Supplementary Table S3. Statistical comparison of VAF and DP between variant callers for shared variants. Wilcoxon rank-sum tests were used for pairwise comparisons, and Kruskal-
Wallis tests were applied when more than two groups were analyzed.

Comparison	Metric	Test Type	Test Statistic	*p-value**
Mutect2 vs. Strelka2	VAF	Wilcoxon	W = 8236	0.0929
Strelka2 vs. FreeBayes	VAF	Wilcoxon	W = 4385	0.9306
Mutect2 vs. FreeBayes	VAF	—	—	—
All Callers (Mutect2, Strelka2, FreeBayes)	VAF	Kruskal-Wallis	$\chi^2 = 0.035$	0.9828
Mutect2 vs. Strelka2	DP	Wilcoxon	W = 7150.5	0.7556
Strelka2 vs. FreeBayes	DP	Wilcoxon	W = 4216.5	0.5900
Mutect2 vs. FreeBayes	DP	—	—	—
All Callers (Mutect2, Strelka2, FreeBayes)	DP	Kruskal-Wallis	$\chi^2 = 0.129$	0.9374

*p-values < 0.05 were considered statistically significant.

Supplementary Table S4. (a) Descriptive statistics for SNVs by ensemble group: sample size (*n*), median and IQR of VAF and DP, range (min–max), and median log10(DP). (b) Pairwise Wilcoxon tests for VAF (two-sided; BH-adjusted *p*-values) with the overall Kruskal–Wallis *p*-value. (c) Pairwise Wilcoxon tests for log10(DP) (two-sided; BH-adjusted *p*-values) with the overall Kruskal–Wallis *p*-value. Statistical significance was defined as $p < 0.05$.

a

Group	n	median VAF	IQR VAF	min VAF	max VAF	median DP	IQR DP	min DP	max DP	median log10(DP)
**Mutect2 only**	162	0.0495	0.044	0.009493	0.375	104	124.5	7	1368	2.017033339
**Strelka2 only**	1084	0.033333333	0.035748792	0.004739336	1	129	93	5	1080	2.11058971
**FreeBayes only**	1196	0.127074549	0.172753203	9.98003992015968E-04	1	242	446.5	8	6319	2.383815366
**Mutect2∩Strelka2**	25	0.030612245	0.010582011	0.013173653	0.157894737	169	140	19	835	2.227886705
**Mutect2∩Strelka2∩SomaticSeq**	236	0.167020374	0.281103128	0.011695906	0.657276995	228.5	304	27	1671	2.358876847
**Strelka2∩SomaticSeq**	36	0.04889684	0.027899386	0.019292605	0.422222222	115	108.5	50	388	2.060632153

b

Group	FreeBayes only	Mutect2 only	Mutect2∩Strelka2	Mutect2∩Strelka2∩SomaticSeq	Strelka2 only	Strelka2∩SomaticSeq
**FreeBayes only**	1	2.13017885314272E-27	1.46544511321197E-10	0.051476126	8.89129806774391E-106	1.08466063291743E-06
**Mutect2 only**	2.13017885314272E-27	1	4.42074052923233E-04	2.11760939288771E-22	1.43605931719456E-04	0.511803777
**Mutect2∩Strelka2**	1.46544511321197E-10	4.42074052923233E-04	1	5.5967555123174E-11	0.240176659	4.41387400350128E-05
**Mutect2∩Strelka2∩SomaticSeq**	0.051476126	2.11760939288771E-22	5.5967555123174E-11	1	6.92807939299996E-45	7.74361697270502E-07
**Strelka2 only**	8.89129806774391E-106	1.43605931719456E-04	0.240176659	6.92807939299996E-45	1	0.00146867
**Strelka2∩SomaticSeq**	1.08466063291743E-06	0.511803777	4.41387400350128E-05	7.74361697270502E-07	0.00146867	1

c

Group	FreeBayes only	Mutect2 only	Mutect2∩Strelka2	Mutect2∩Strelka2∩SomaticSeq	Strelka2 only	Strelka2∩SomaticSeq
**FreeBayes only**	1	3.15979188945278E-14	0.326351413	0.806908625	3.09360421402759E-36	3.40248164250685E-04
**Mutect2 only**	3.15979188945278E-14	1	0.005308088	1.60458328199628E-15	9.11882962428004E-04	0.36292778
**Mutect2∩Strelka2**	0.326351413	0.005308088	1	0.127845213	0.010470281	0.016415351
**Mutect2∩Strelka2∩SomaticSeq**	0.806908625	1.60458328199628E-15	0.127845213	1	6.25744367297423E-26	1.56795028838927E-06
**Strelka2 only**	3.09360421402759E-36	9.11882962428004E-04	0.010470281	6.25744367297423E-26	1	0.326351413
**Strelka2∩SomaticSeq**	3.40248164250685E-04	0.36292778	0.016415351	1.56795028838927E-06	0.326351413	1
