# Supplementary material for: Comparative Evaluation of Mutect2, Strelka2, and FreeBayes for Somatic SNV Detection in Synthetic and Clinical Whole-Exome Sequencing Data
Source: Biomolecules. 2025 Oct 30;15(11):1532. doi: 10.3390/biom15111532 (PMC12650410; doi:10.3390/biom15111532)
Supplement: Supplementary file 1 [file biomolecules-15-01532-s001.zip › Supplementary Figures_round2.pdf]

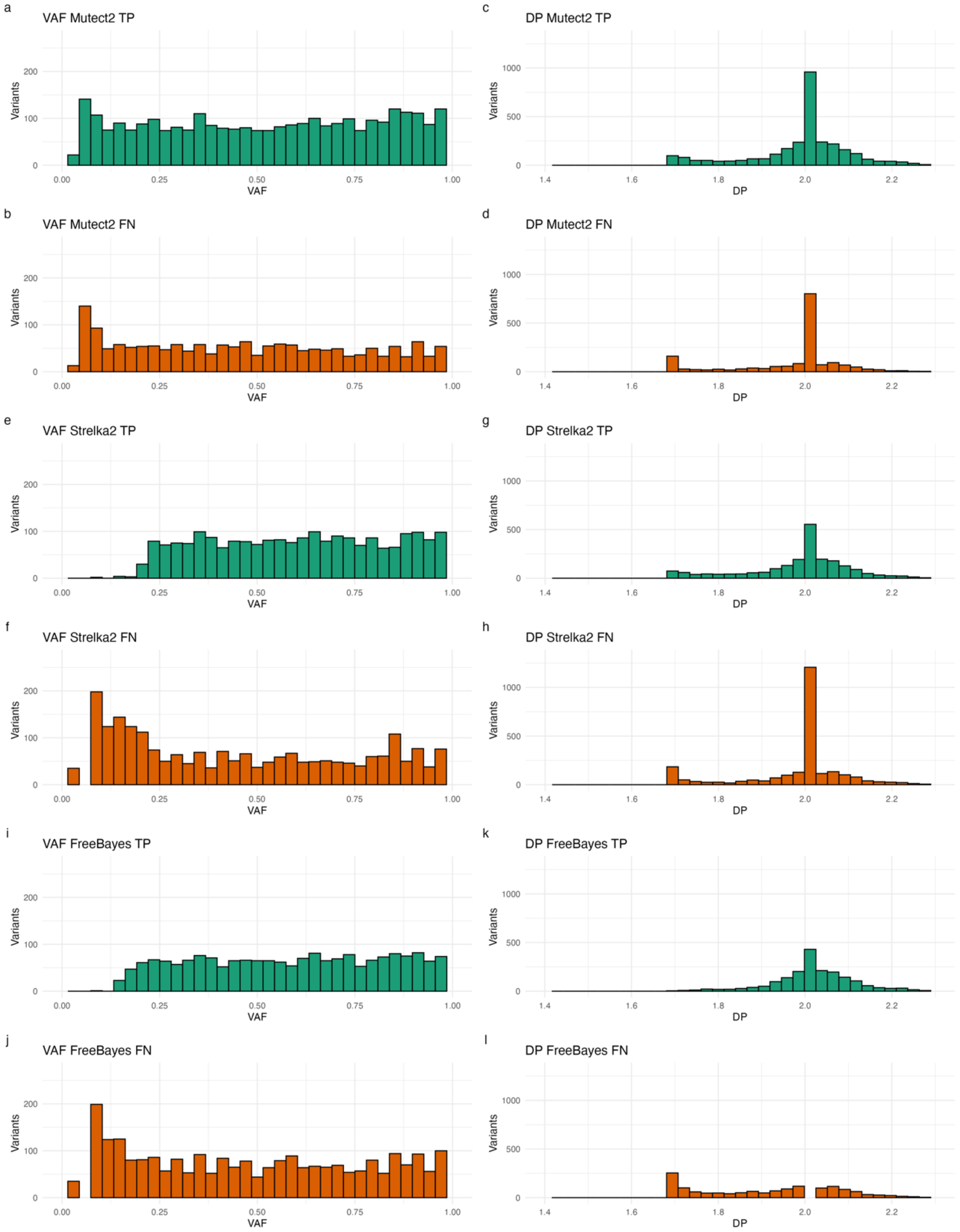

Supplementary Figure S1. Distribution of VAF and log_{10}-transformed sequencing DP of detected and undetected variants for each VCs in synthetic WES dataset. Detected true positives (shown in green) and undetected variants (false negatives, shown in red) are displayed separately.

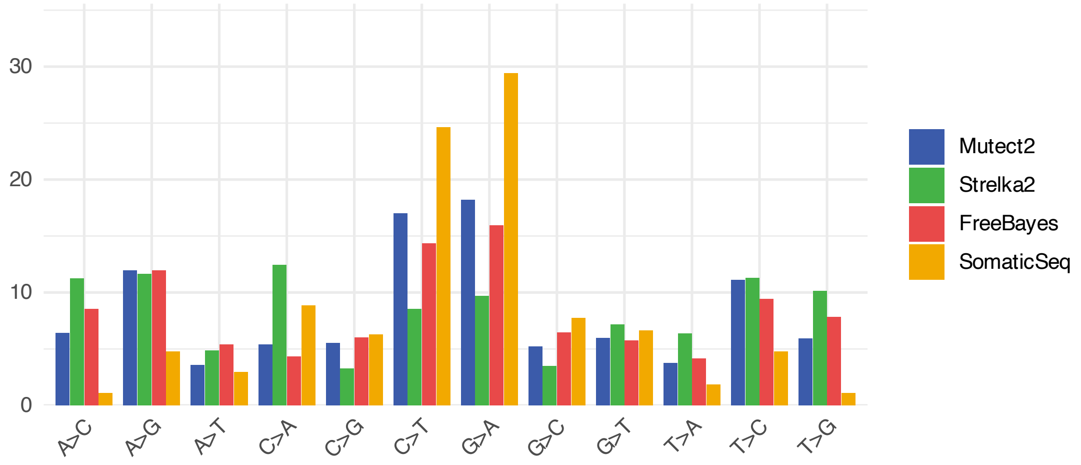

Supplementary Figure S2. Distribution of single-nucleotide substitution types in OC WES data. Substitution spectrum detected independently by Mutect2, Strelka2, FreeBayes, and SomaticSeq consensus variants.

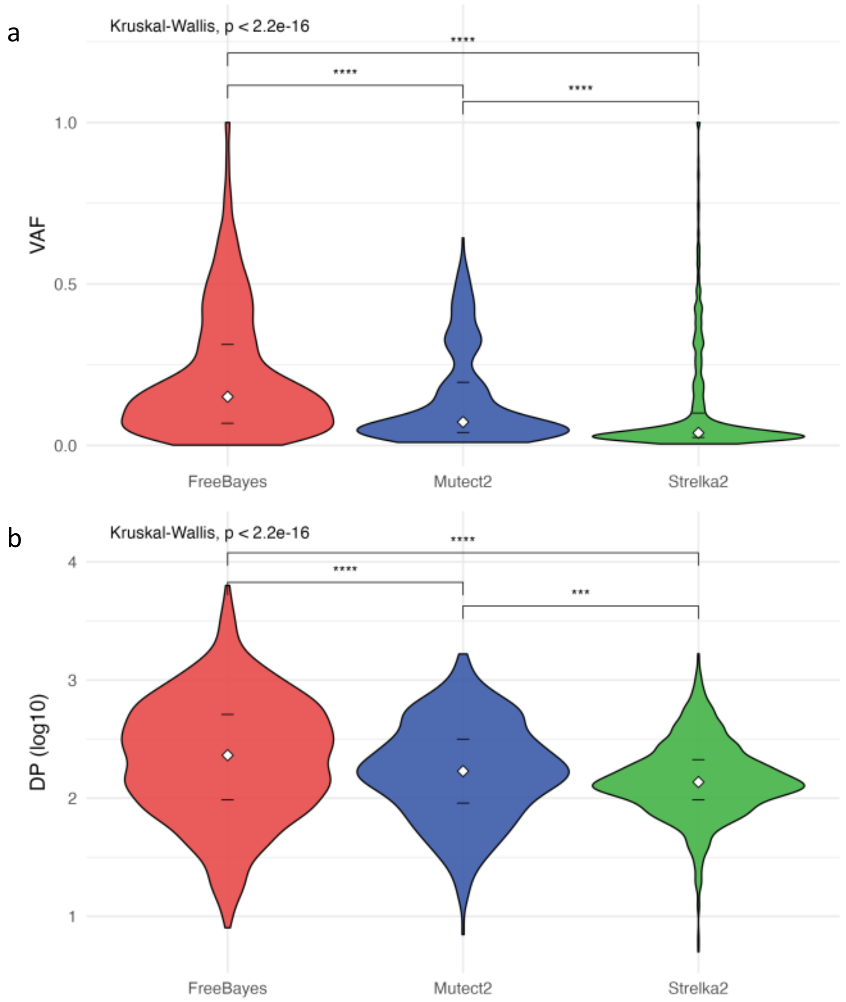

Supplementary Figure S3. Distribution of VAF and DP for SNVs detected by FreeBayes, Mutect2, and Strelka2 in OC WES data. The white diamond indicates the median, while the horizontal black lines represent the first (Q1) and third (Q3) quartiles. Horizontal bars denote pairwise Wilcoxon rank-sum tests. Kruskal-Wallis test p-values are shown in the upper left corner of each panel. Significance codes: * $p < 0.05$, ** $p < 0.01$, *** $p < 0.001$, **** $p < 0.0001$.

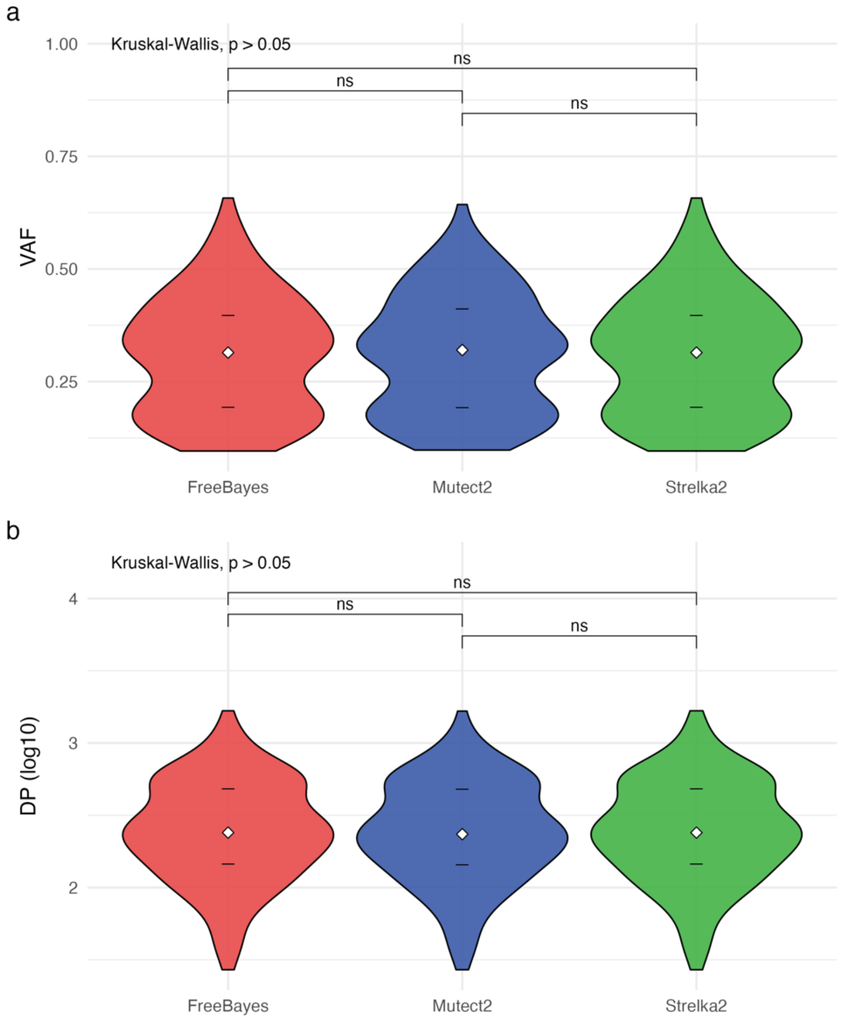

Supplementary Figure S4. Distribution of VAF and DP for shared SNVs detected by FreeBayes, Mutect2, and Strelka2 in OC WES data. The white diamond indicates the median, while the horizontal black lines represent the first (Q1) and third (Q3) quartiles. Horizontal bars denote pairwise Wilcoxon rank-sum tests. Kruskal-Wallis test p-values are shown in the upper left corner of each panel. Significance codes: ns = not significant, * $p < 0.05$, ** $p < 0.01$, *** $p < 0.001$, **** $p < 0.0001$.

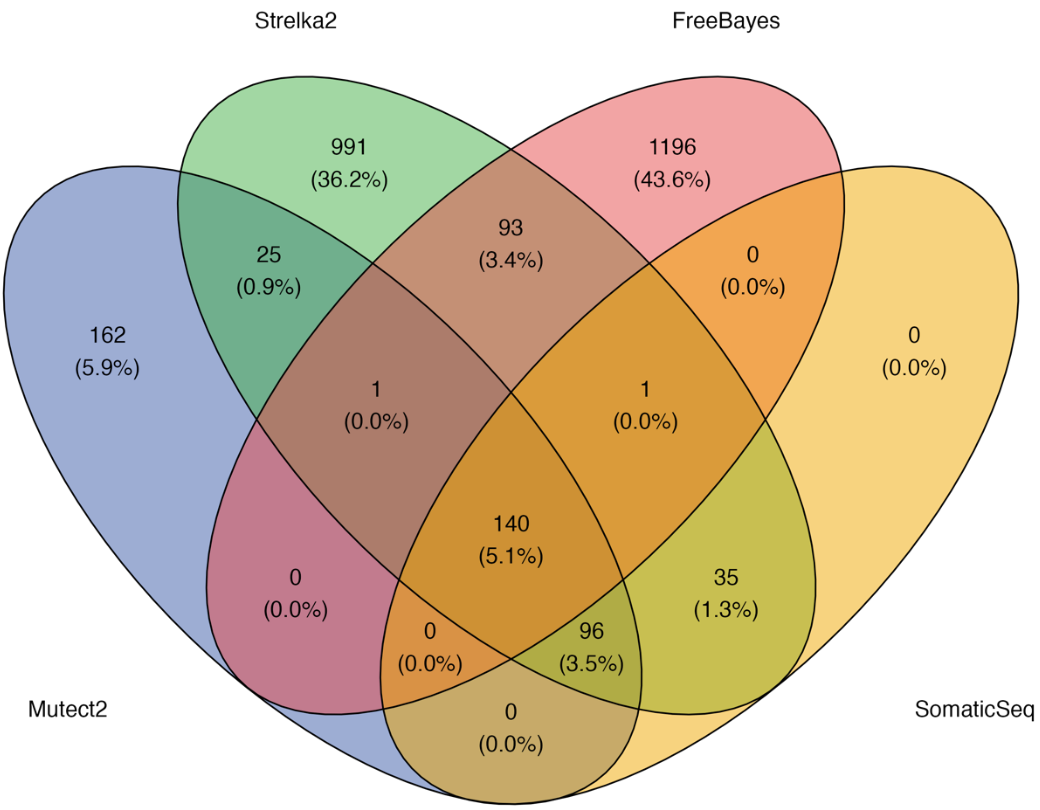

Supplementary Figure S5. Four-set Venn diagram by Mutect2, Strelka2, FreeBayes, and SomaticSeq (consensus mode). Each region shows the count and percentage of variants relative to the union across callers. Colors correspond to the four callsets (Mutect2: blue; Strelka2: green; FreeBayes: red; SomaticSeq: orange). Only SNVs are included.

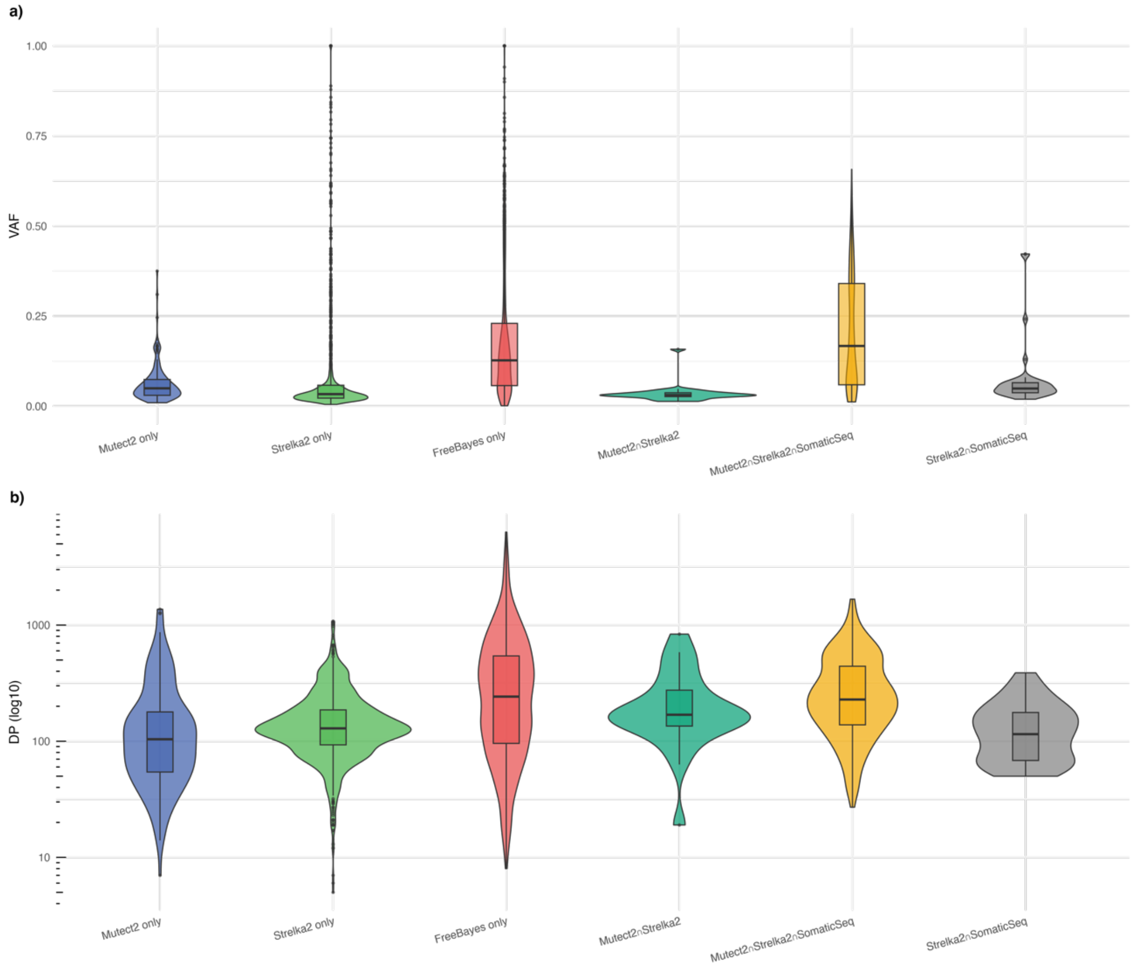

Supplementary Figure S6. Violin plots summarizing (a) VAF and (b) DP (log10 scale) for SNVs partitioned into six groups: Mutect2-only, Strelka2-only, FreeBayes-only, Mutect2∩Strelka2-only, Mutect2∩Strelka2∩SomaticSeq, and Strelka2∩SomaticSeq-only. Boxes indicate the median and interquartile range within each violin.
